# Comparison of Fork‐tip and Franseen needles for endoscopic ultrasound‐guided fine‐needle biopsy in pancreatic solid lesions: A propensity‐matched analysis

**DOI:** 10.1002/deo2.147

**Published:** 2022-06-28

**Authors:** Akashi Fujita, Shomei Ryozawa, Yuki Tanisaka, Tomoya Ogawa, Yoichi Saito, Hiromune Katsuda, Kazuya Miyaguchi, Masanori Yasuda, Ryuichiro Araki, Yumi Mashimo, Tomoaki Tashima, Yuya Nakano, Rie Terada, Ryuhei Jinushi, Masafumi Mizuide

**Affiliations:** ^1^ Department of Gastroenterology Saitama Medical University International Medical Center Saitama Japan; ^2^ Department of Pathology Saitama Medical University International Medical Center Saitama Japan; ^3^ Community Health Science Center Saitama Medical University Saitama Japan

**Keywords:** endoscopic ultrasound, endoscopic ultrasound‐guided fine‐needle biopsy, Franseen needle, Fork‐tip needle, histology

## Abstract

**Objectives:**

There is no unanimity regarding the most appropriate needle to use for an endoscopic ultrasound‐guided fine‐needle biopsy (EUS‐FNB). To date, new types of FNB needles have been designed, including the Fork‐tip and Franseen needles. This study primarily aimed to compare the diagnostic accuracy and histological quality between the use of the Franseen and Fork‐tip needles in EUS‐FNB for solid pancreatic lesions.

**Materials and methods:**

We retrospectively analyzed 147 patients at our center for solid pancreatic lesions, 75 of whom underwent EUS‐FNB using a 22‐G Franseen needle, and 72 using a 22‐G Fork‐tip needle, from December 2019 to September 2021. The present study conducted a propensity‐matched analysis and confounder adjustment.

**Results:**

The diagnostic accuracy of the Fork‐tip group (93.3%, 42/45) was the same as that of the Franseen group. For the core tissue and blood scores, no significant difference was observed (*p* = 0.58, 0.25) between the two groups. The rate of changes in the operator from that of a trainee to an expert was less in the Fork‐tip group (4.4%, 2/45) than in the Franseen group (15.6%, 7/45), but not significantly different (*p* = 0.16).

**Conclusions:**

In both groups, the diagnostic accuracy and histological quality were not significantly different. Additionally, there were no significant differences in the rate of operator changes. As both needles are useful, the choice of using either of them is equally good.

## INTRODUCTION

Endoscopic ultrasound‐guided fine‐needle aspiration (EUS‐FNA) was first reported in 1992.^1^ Today, this technique is recognized for being the crucial modality used in the pathological diagnosis of pancreatic lesions.^2^, ^3^ In most cases, cytological or histological evaluation of small tissue fragments is sufficient to distinguish between malignant and benign lesions.^4^ However, the amount of core tissue acquired using EUS‐FNA is mostly insufficient for additional diagnoses. Besides conventional diagnosis, the use of other techniques, such as genetic diagnosis and anticancer drug sensitivity measurement, is therefore limited with this limited amount of tissue.^5^

There are several different opinions on the choice of needle and no definitive recommendation. In EUS‐FNA, a 19G needle is useful for tissue diagnosis because it provides sufficient specimens for immunostaining, but this size has a large puncture resistance, which increases the difficulty of the procedure.^6^ Recently, there have been increasing reports on the usefulness of needles, such as the fine‐needle biopsy (FNB) needles, which are mainly intended for core tissue acquisition, as their designs continue to be advanced.^7^ With EUS‐FNB, more information regarding tissue structure and better sample yields may be obtained, which may enable further analysis as well as improve diagnostic accuracy.^8^

Currently, various FNB needles exist, and their usefulness has been extensively reported.^9^, ^10^ To date, new types of FNB needles have been designed for use, including the Fork‐tip and Franseen needles. The Fork‐tip is characterized by two sharp tips on opposite sides of the lumen,^11^ and the Franseen needles are characterized by three symmetric cutting tips.^12^ However, there is no consensus regarding the best needles. Hence, the current study aimed to assess the diagnostic accuracy and histological quality of EUS‐FNB for solid pancreatic lesions using the 22‐G Franseen and Fork‐tip needles.

## MATERIALS AND METHODS

### Patients

All patients who underwent EUS‐FNB for solid pancreatic lesions from December 2019 to September 2021 were retrospectively evaluated at our center; 147 patients were analyzed, of whom 75 (Franseen group) underwent EUS‐FNB with the Franseen needle (22‐G) between December 2019 and November 2020 and 72 (Fork‐tip group) with Fork‐tip needle (22‐G) between December 2020 and September 2021.

### Study definition and measurements

The following information was obtained from the patients’ electronic medical records: age, sex, tumor size, tumor site, puncture site, endoscopist's experience with the procedure, and the name of the final diagnosis. The definition of the final diagnosis was determined by considering the histological diagnosis at the time of surgery for patients who underwent surgery and the clinical course after 6 months for patients who did not undergo surgery. Pancreatic adenocarcinoma, pancreatic neuroendocrine tumor (NET), pancreatic carcinomas other than adenocarcinomas, and solid pseudopapillary neoplasms were defined as malignant lesions. Non‐neoplastic lesions, such as focal pancreatitis, were defined as benign lesions if there were no malignant findings on histological examination and no increase in size after 6 months of follow‐up.

**FIGURE 1:**
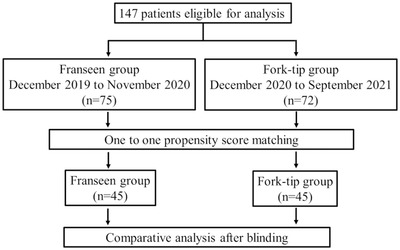
Diagram of the study design

We used the propensity‐matched analysis and adjusted the confounders in all patients. After the propensity‐matched analysis, we analyzed the patients’ electronic medical records (Figure 1). This study primarily aimed to compare the diagnostic accuracy and histological quality of EUS‐FNB in pancreatic solid lesions between the use of the Franseen and Fork‐tip needles, and secondly to evaluate the rate of operator changes. This study was approved by the ethics review board at Saitama Medical University International Medical Center (No. 18‐253), which complied with the Declaration of Helsinki as revised in Brazil in 2013. All patients provided written informed consent for EUS‐FNB.

### Procedures

A convex linear‐array echoendoscope (GF‐UCT260; Olympus Optical, Tokyo, Japan) combined with an ultrasound system (EU‐ME2 Premier Plus; Olympus Optical) was used for EUS‐FNB procedures. During EUS, intravenous midazolam and pethidine hydrochloride were administered for giving sedation. The vasculature from regional and collateral regions was excluded from the puncture route, and the target lesion was punctured. The stylet was then withdrawn, and incessant suction was performed using a syringe (20 ml).

Afterward, within the lesion, approximately 20–30 rapid strokes were performed, following which the suction was released, and the needle was removed. Additionally, aspirated samples were smeared onto glass slides via stylet insertion, and air pressure was applied. The samples were examined visually for white coloration and then fixed in formalin for histological examination.

Given that the rapid on‐site cytologic examination was not possible in our hospital, we repeated the puncture while discussing with the cytology technician until it was determined that sufficient samples for histopathology and immunostaining were visually available.

**FIGURE 2:**
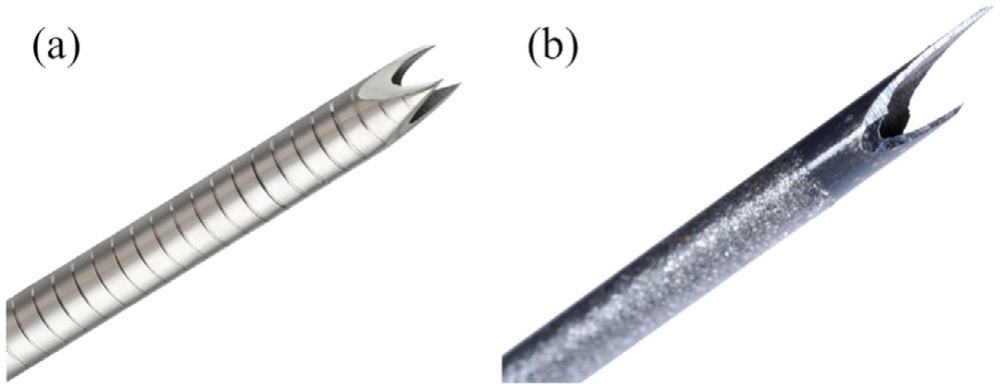
(a) Franseen needle with three symmetric cutting tips for an endoscopic ultrasound‐guided fine‐needle biopsy. (b) Fork‐tip needle with two sharp tips on the opposite side of the lumen

For the Franseen group between December 2019 and November 2020, the Acquire Franseen needle (Boston Scientific, Marlborough, MA, USA) was primarily employed, and for the Fork‐tip group, the SharkCore Fork‐tip needle (Medtronic, Newton, Mass and Covidien, Dublin, Ireland) was used thereafter (Figure 2a,b). Six endoscopists (four trainees and two experts on EUS‐FNB) performed the procedures. The four trainee endoscopists had adequate experience, conducting more than 1000 regular esophagogastroduodenoscopies (EGDs), 20 EUS procedures, and 500 colonoscopies, and were also involved in assisting the expert endoscopists for 20 EUS‐FNA/FNB procedures. Meanwhile, the two expert endoscopists had regularly been performing regular EGD, colonoscopy, and EUS procedures with more than 50 EUS‐FNA/FNB procedures before the beginning of this study. Two trainees executed the procedures in each group but did not perform the procedures across both groups. Trainees executed the EUS‐FNB in all cases. An expert was requested if the procedure was difficult to complete. The trainee operator was replaced with an expert if he/she could not visualize or puncture the target. Technical success was defined as the successful puncture to the target.

### Histologic evaluation

**FIGURE 3:**
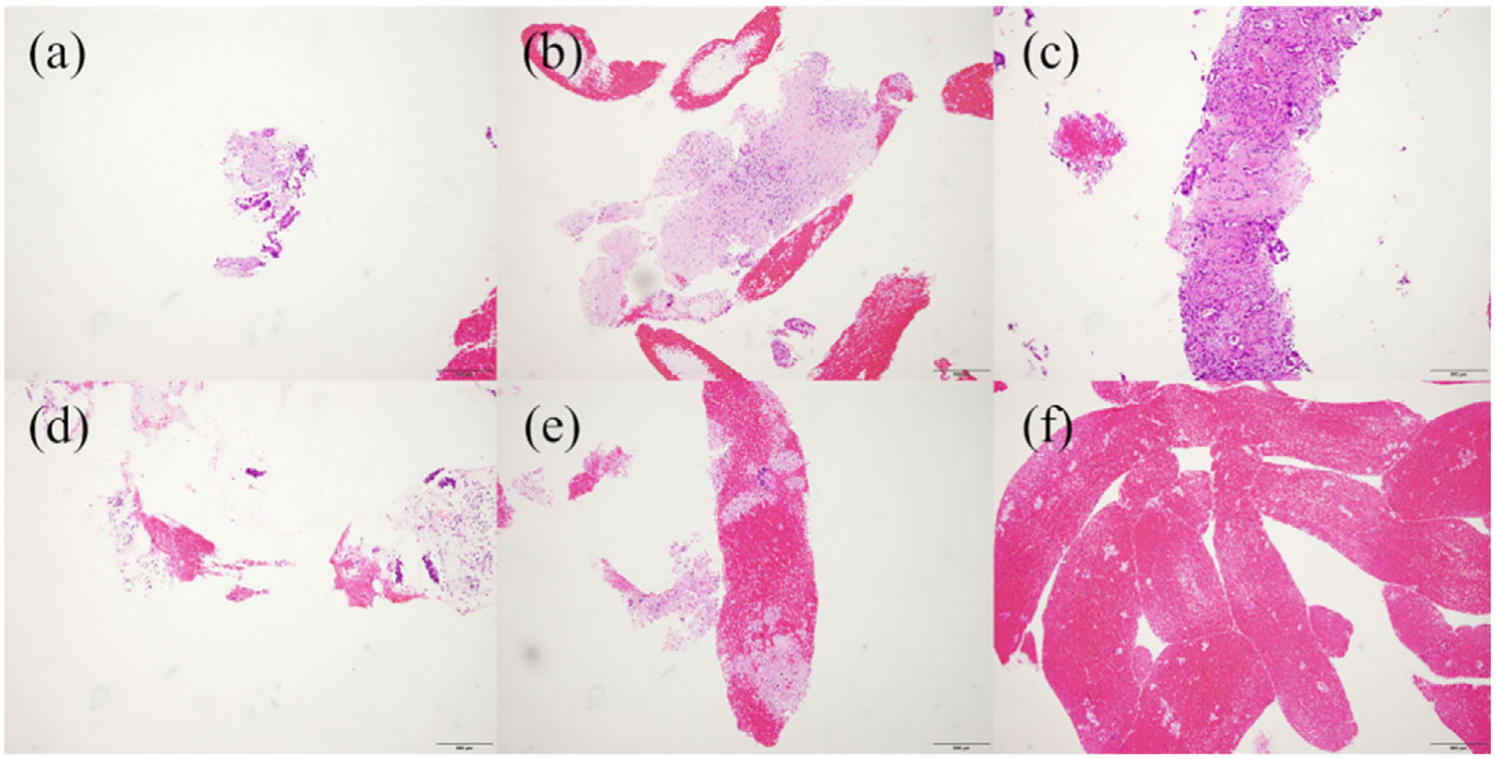
(a) Amount of core tissue score 2 (a tissue fragment). (b) Amount of core tissue score 3 (a small histological core tissue, < × 10 objective). (c) Amount of core tissue score 4 (a large histological core tissue, > × 10 objective). (d) Amount of blood score 1 (none–few). (e) Amount of blood score 2 (moderate). (f) Amount of blood score 3 (high)

The specimens collected via EUS‐FNB were smeared onto glass slides. The specimens obtained were checked for sufficiency and were then preserved in neutral buffered formalin (10%) followed by paraffin embedding. For histological examination, specimen sections were cut into 4 μm thick serial sections for hematoxylin and eosin, followed by immunostaining, as needed. In this study, only the histological diagnoses were analyzed by two pathologists who were blinded regarding the type of needle to be used. Analysis of the tissue specimens was conducted using the volume of the core tissue (scores 1–4) and the level of blood contamination (scores 1–3):^4^, ^13^ 1 = no material, 2 = a tissue fragment, 3 = a small histological core tissue < ×10 objective, and 4 = a large histological core tissue > ×10 objective for the core tissue volume and 1 (few), 2 (moderate), and 3 (high) for the blood contamination volume (Figure 3).

### Statistical analysis

Categorical variables were expressed as absolute (*n*) and relative (%) frequencies and were compared using Fisher's exact test. To compare normally distributed continuous data, a two‐sample *t*‐test was conducted, and the Mann–Whitney test was performed if normality could not be demonstrated. To create a propensity score‐matched cohort, we attempted to match each patient from the Franseen group with a patient from the Fork‐tip group by using an optimal matching technique; this was aimed to reduce the bias in the selection and the possible potential confounding.

A propensity score ranging from 0 to 1 was created using the six variables including sex, age, puncture route, and tumor size, site, and type (lesions requiring or not requiring immunostaining), which could affect the outcome, and it was performed via logistic regression. *p* < 0.05 values were considered significant. To calculate the statistical data, the SAS JMP version 14.3.0 (SAS Institute, Cary, NC, USA) and EZR version 1.54 software (Saitama Medical Center, Jichi Medical University, Saitama, Japan) were applied.

## RESULTS

### Patient characteristics

As mentioned, we analyzed 147 patients who had undergone EUS‐FNB for pancreatic solid lesions. In the present study, a propensity‐matched analysis and confounder adjustment were conducted. Initially, the Franseen and Fork‐tip groups had 75 and 72 cases, respectively. After propensity‐matched analysis, each group was adjusted to 45 cases. Table 1 lists the clinical features of each propensity‐matched patient group. The results revealed that sex, age, route for puncture, and tumor size, site, and tumor type were not different (significant, *p‐*value) between the Franseen and Fork‐tip groups.

**TABLE 1:** Clinical features of patients

	**All patients**			**Propensity‐matched patients**		
	**Franseen group**	**Fork‐tip group**	***p***	**Franseen group**	**Fork‐tip group**	***p***
Sex, male/female	41/34	35/37	0.51	22/23	19/26	0.67
Age (years), median (IQR)	71.0 (66.5–76.0)	70.0 (63.8–76.3)	0.62	71.0 (65.0–74.0)	70.0 (64.0–76.0)	0.74
Tumor size (mm), median (IQR)	26.7 (22.3–31.5)	25.0 (22.8–30.1)	0.37	26.7 (21.9–31.4)	25.0 (22.6–30.0)	0.31
Pancreatic head/ body or tail	16/59	37/35	<0.001	16/29	14/31	0.82
Transgastric/ transduodenal	60/15	36/36	<0.001	30/15	32/13	0.82
Lesions requiring/ not requiring immunostaining	4/71	6/66	0.53	3/42	2/43	>0.99

Abbreviations: IQR, interquartile range; *n*, number of lesions.

### Final diagnosis

From the findings, the most common final diagnosis was adenocarcinoma (72 patients), which was followed by a benign lesion (13 patients), and finally, the NET (five patients) (Table 2).

**TABLE 2:** Final diagnosis of propensity‐matched patients

**Final diagnosis**	**Franseen group, *n* (%)**	**Fork‐tip, group *n* (%)**
Adenocarcinoma	35 (77.8%)	37 (82.2%)
Benign lesions (focal pancreatitis)	7 (15.6%)	6 (13.3%)
NET	3 (6.7%)	2 (4.4%)
Overall	45 (100%)	45 (100%)

Abbreviations: *n*, number of lesions; NET, neuroendocrine tumor.

### Procedure outcome

In this study, no adverse events during the procedure were encountered. For both groups, the number of punctures, time of the procedure, and technical success rate were not significantly different.

The rate of change in the operator (from trainee to an expert) was less in the Fork‐tip group (4.4%, 2/45) than in the Franseen group (15.6%, 7/45), but it was not significantly different (*p* = 0.16). The reasons for operator changes were as follows: In the Franseen group, there were four cases of difficulty in adjusting the puncture route from the second part of the duodenum, two cases of difficulty in penetrating the gastric wall from the stomach, and one case in which the target could not be visualized using EUS image. In contrast, in the Fork‐tip group, there were only two cases in which the operator was changed because of difficulty in visualizing the target, and there were no cases in which a puncture was difficult.

The diagnostic accuracy of the Fork‐tip group (93.3%, 42/45) was the same as that of the Franseen group. The outcomes of the two groups were similar for the distinction between benign and malignant lesions (Table 3).

**TABLE 3:** Comparison of procedure outcome and histological material between the Franseen and Fork‐tip groups

	**Propensity‐matched patients**		
	**Franseen group**	**Fork‐tip group**	***p***
Number of punctures, median (IQR)	2 (2–3)	2 (2–3)	0.67
Procedure time (min), median (IQR)	24.0 (19.0–32.0)	26.0 (20.0–30.0)	0.78
Technical success, *n* (%)	45/45 (100%)	45/45 (100%)	>0.99
Change from trainee to expert	7/45 (15.6%)	2/45 (4.4%)	0.16
Diagnostic accuracy, *n* (%)	42/45 (93.3%)	42/45 (93.3%)	>0.99
Sensitivity	92.1% (35/38)	92.3% (36/39)	>0.99
Specificity	100% (7/7)	100% (6/6)	
Positive predictive value	100% (35/35)	100% (36/36)	
Negative predictive value	70% (7/10)	66.7% (6/9)	
Adverse events	0/45 (0%)	0/45 (0%)	
Amount of core tissue score 1	0	0	
Score 2	11	8	
Score 3	20	22	
Score 4	14	15	
Median (IQR)	3 (3–4)	3 (3–4)	0.58
Degree of blood contamination score 1	5	4	
Score 2	20	28	
Score 3	20	13	
Median (IQR)	2 (2–3)	2 (2–3)	0.25

Abbreviations: IQR, interquartile range; *n*, number of lesions.

### Comparison of histological material

For the core tissue score concerning the amount of tissue, scores 3 and 4 accounted for a large percentage of the score, and between the two groups, no significant difference was observed (Franseen group = 34/45 vs. Fork‐tip group = 37/45, *p* = 0.58). Regarding the blood scores, most scores were 2 and 3, with no significant difference (Franseen group = 40/45 vs. Fork‐tip group = 41/45, *p* = 0.25; Table 3).

## DISCUSSION

Several studies have compared FNB with FNA in recent years.^9^, ^14^ We previously evaluated the usefulness of EUS‐FNB using the Franseen needle (22‐G), in comparison with EUS‐FNA conducted with a conventional needle (22‐G) for the diagnosis of pancreatic diseases.^5^ The Franseen needle group demonstrated a diagnostic accuracy of >90% with fewer punctures compared with the FNA needle group (median 2 vs. 3, *p* = 0.028). This could be due to the bigger amount of tissue that could be collected using a Franseen needle, and the white tissue specimens were easier to recognize visually. Acquiring more samples can improve the precision of diagnosis for pathologists, and also, the number of punctures can be reduced. Moreover, results have been published from a randomized controlled trial that compared the outcomes in solid pancreatic lesions for the 22‐G reverse bevel‐designed FNB with the 22‐G FNA needle.^15^ The outcomes for sampling had demonstrated a higher adequate sample rate in the FNB group than in the FNA group (100% vs. 90%, *p* = 0.02), and the values were significant. Also, the samples attained using FNB needles were of better histological quality while requiring a smaller number of needle passes.

**FIGURE 4:**
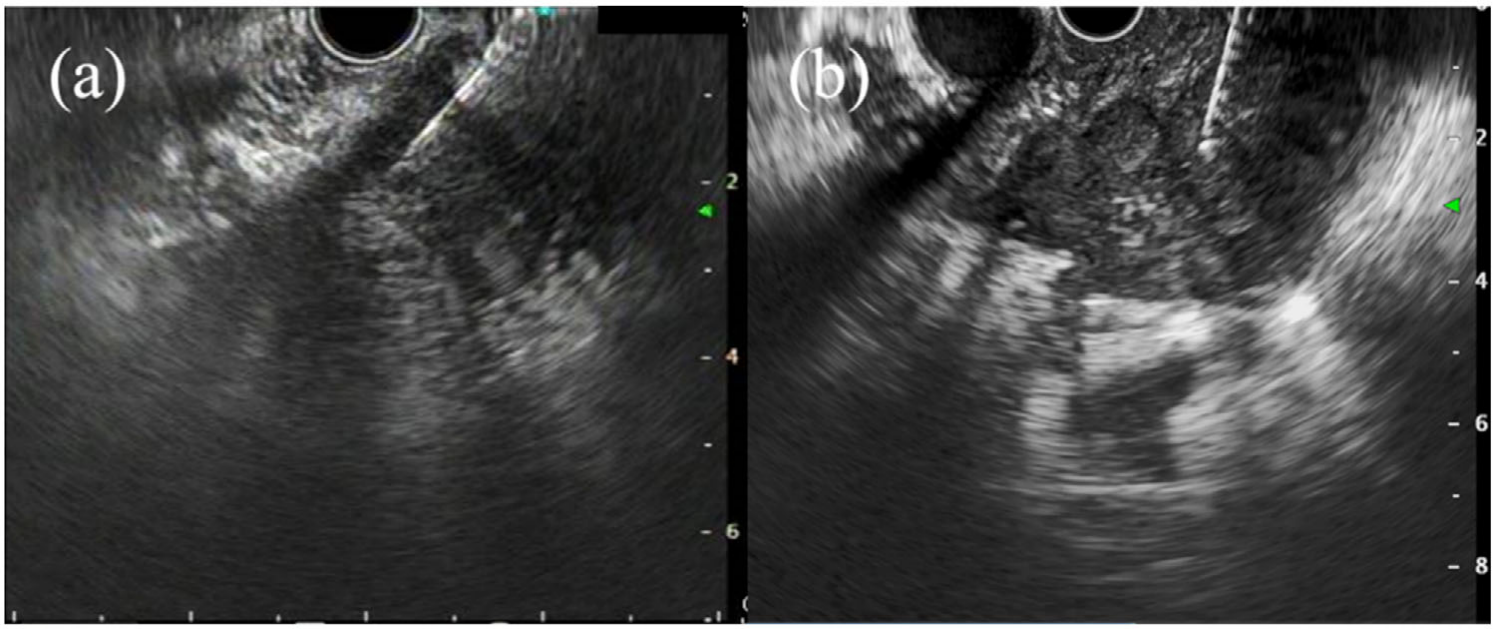
(a) Endoscopic ultrasound‐guided fine‐needle biopsy from the second part of the duodenum using the Franseen needle. (b) Endoscopic ultrasound‐guided fine‐needle biopsy from the second part of the duodenum using the Fork‐tip needle.

Larger histological sample volumes by FNB would facilitate both histological diagnosis and further analysis, such as the assessment of molecular expression or DNA sequencing. Recently, Olaparib, which is the poly (adenosine diphosphate‐ribose) polymerase inhibitor, was shown to be effective in phase III trials for metastatic pancreatic cancer with germline BRCA mutations.^16^ Other molecular targeted drugs, such as entrectinib or pembrolizumab, are also being used. The need for precision medicine is steadily spreading in pancreatic cancer.^17^, ^18^ For personalized medical treatments, the choice of an FNB needle may be advantageous. However, as mentioned before, there is still no answer regarding which FNB needle will be recommended. A meta‐analysis on the most recent needles, Franseen and Fork‐tip, has been published.^19^ The analysis included approximately 21 studies conducted on 1632 patients. The total pooled diagnostic yield was 92.8% (95% CI 85.3–96.6, *I* ^2^ = 73.1) using a Fork‐tip needle and 92.7% (95% CI 86.4–96.2, *I* ^2^ = 88.4) using a Franseen needle; there was no statistical difference between the two needle groups (*p* = 0.98). Similarly, in this study, in both groups, the diagnostic accuracy and the overall outcomes of the other procedures were not significantly different. Nevertheless, it is worth noting that the rate of changes in the operator from that of a trainee to an expert was less in the Fork‐tip group (4.4%, 2/45) than in the Franseen group (15.6%, 7/45; *p* = 0.16); however, no significant difference was observed. Fork‐tip needles are made of stainless steel. The angle of a puncture could be easily adjusted because the material is soft and flexible, particularly when puncturing from the second part of the duodenum. Conversely, the Franseen needle used in this study is made of cobalt‐chromium, which is harder than stainless steel; thus, it is more difficult to adjust the angle.^8^ Therefore, to achieve the proper puncture, when the Franseen needle is used, it is necessary to pull back the scope more compared with the Fork‐tip needle (Figure 4). Additionally, the three tips of the Franseen needle sometimes create resistance when puncturing the gastrointestinal wall, resulting in poor penetration. For these reasons, it can sometimes be difficult to perform procedures using the Franseen needle. The Franseen needle may be an option when multiple punctures are required to obtain a large sample volume because the needle material is hard, and it does not easily bend even after repeated punctures. As described above, the appropriate needle must be selected according to the situation. To the best of our knowledge, this is the first report to evaluate the rate of operator changes in addition to the diagnostic accuracy and histological quality of these needles.

There were several limitations to this study that should be considered while interpreting the data. The probable potential effects of using different types of needles at different time points cannot be ruled out. This may affect the results due to the different learning curves of the operators. Although we adjusted for background factors in the propensity‐matched analysis, the low number of patients is a limitation. Additionally, we retrospectively collected all data from a single study center. Procedures were performed by six endosonographers; hence, there was a risk of heterogeneity among the operators. A future prospective study based on a larger number of cases will be required. Furthermore, in this study, only the continuous aspiration techniques with a 20‐ml syringe were performed. Continuous aspiration with the stylet technique (also called the stylet slow‐pull technique) has been previously described to help avoid contamination of blood in the collected specimens.^20^, ^21^, ^22^

In conclusion, in both groups, diagnostic accuracy and histological quality were not significantly different. In addition, there is no significant difference in the rate of operator changes. As both needles are useful, the choice of using either of them is equally good.

## CONFLICT OF INTEREST

The authors declare no conflict of interest.

## FUNDING INFORMATION

None

## Data Availability

The datasets in the present study and the analysis of the data will be available from the corresponding author upon reasonable request.
